# Preface of Special Issue “Cares in the Age of Communication: Health Education and Healthy Lifestyles”: Social Media and Health Communication in a Pandemic?

**DOI:** 10.3390/ejihpe10020042

**Published:** 2020-04-10

**Authors:** Iván Herrera-Peco, Julio C. de la Torre-Montero

**Affiliations:** 1 Health Sciences College, Alfonso X el Sabio University, 28691 Madrid, Spain; 2 San Juan de Dios School of Nursing and Physical Therapy, Comillas Pontifical University, 28015 Madrid, Spain

## 1. Introduction

In the midst and the mist of the Covid-19 outbreak, we are living in the age of global communication in a hyperconnected society in which the transmissions channels between people have been changed very clearly due to both the internet itself in general and social networks in particular [1]. These represent very powerful tools when searching for health information or collecting such information [2], but they also exemplify a clear way for patients to share information [3,4] and healthcare professionals to meet the needs and concerns of patients and caregivers [1,4,5].

Directly associated with the use of social networks and the internet, it is important to highlight a series of data that can show that this situation is of great importance. In 2016 there were more than 7000 million mobile users, and this number has also increased globally year after year, while the use of the internet also increased globally from 6.5% to 43% between 2000 and 2015 [6].

Focusing on the case of Spain, it is observed that during 2019, 91.4% of Spanish households have access to the internet, while 90.7% of the population between 16 and 74 years have used the internet weekly [7]. Of all these internet users, 96.9% say they have used a mobile phone to access the internet and check its contents [7]. Likewise, and to emphasize the importance of social networks, it was indicated that 64.6% of internet users use the internet to consult social networks, and in addition, 72.1% of women and 60.3% of men use the internet to search for health-related information [7]. These data, limited only to Spain, show the large amount of information that is generated as well as the existing contact networks involving the internet.

However, with regard to social media, it is important to understand that it has been associated with personal interactions, a situation that has been supported by the great technological development that occurred at the beginning of the 21st century, since it has de facto changed our way of communicating, exchanging information, impressions, etc. [8]. Today various forms of virtual social media, such as Facebook, Twitter, Instagram, etc., provide some of the most powerful forms of communication between people around the world [1]. 

In the light of the above, it has to be concluded that we can now see an increasing flow of data and information related to health, while the population is increasingly co-responsible, together with health professionals, for their own health and quality of life [9] by generating relevant information and data through interactions involving the internet and social networks, which can potentially be used by researchers [9]. However, it is necessary not to forget that social media could be the fastest way to transfer knowledge of current practices to healthcare professionals and other people that need to hear about it during natural disasters, pandemics, etc. [10]. 

Twitter is a social network service that was created in 2006 [11,12], and in 2016 it had more than 328 million active users per month [13], while more than 500 million tweets have been released each day by more than 300 million active users [13,14]. This social network is available in more than 33 different languages, and even supports non-Latin characters.

These characteristics facilitating both a rapid exchange of information and an ease of generating both unidirectional and bidirectional contact networks [14], make Twitter a social media of special interest when it comes to disseminating information related to health or scientific information, but also an element to keep in mind when designing and proposing research projects related to, for example, monitoring the flow of information, evaluating the effectiveness of awareness or information dissemination campaigns, etc. [14,15].

## 2. Social Media as Important Tool in Public Health Surveillance

An important role of social media is helping researchers to develop epidemiological studies when health emergencies appear, such as the spread of infectious diseases. Twitter can not only be useful when looking for information about the state of this emergency, but it could also be useful for studying opinions, emotions or the strategies that people are adopting to protect themselves. This information is very useful for governments, public health agencies or researchers in epidemiology and public health [15,16,17]. 

But Twitter is also useful for helping with actions focused on health promotion at a remote level, as the monitoring of physical activity during confinement yields very interesting figures, such as a great decrease in physical activity, which can be recorded through wearable devices [18].

### Epidemiological Information

Twitter is taking advantage of the full potential of its internet and web-based services though the monitoring of various infectious diseases and epidemiological studies such as the H1N1 Flu virus [16], Zika disease [19,20], Dengue virus [21], Ebola [15,22] or even COVID-19 [10,23]. 

Because Twitter is a free service that enables millions of users to send short messages, it provides useful information that allows, for example, for tracking rapidly evolving public opinion regarding H1N1 or swine flu. Results have shown that disease estimates using these conversations on Twitter accurately correspond to disease levels reported by authorities [16].

On the other hand, Miller et al.’s study [19] focused on specific social concerns related to the prevention, transmission, symptoms and treatment of the Zika virus, for which they collected 1,234,605 tweets discussing specific aspects of Zika or that contained associated misinformation. With this information, five topics were created for each category (prevention, transmission, treatment and vaccine in development), which future studies will be able to use to automatically detect erroneous information using Twitter and thus allow well-directed and timely interventions [19].

Another example of social media use could be the experience of Sousa et al. [21]. They developed a mobile application (*VazaDengue*) to prevent and combat mosquito-borne diseases, in which citizens report mosquito breeding sites and cases of dengue fever. The tweets are then classified, and the relevant ones are tagged as reports, and the reports are then made available to the community and health agencies. This study shows that health workers tend to agree with the relevance of classified tweets. Furthermore, tweeting is likely to be effective for monitoring diseases in large cities.

## 3. Limitations of Social Media

In regard to the gathering of scientific information, the main risk from social media could be the dissemination of misinformation [10], which could occur not only when accessing non-peer reviewed materials but also when using peer-reviewed resources. People without adequate scientific formation can misinterpret this information and create fake news [24]. These kinds of fake news spread through a large number of users and modify perceptions and understandings of events and even facts [25] because they are based on scientific communication. Of course, one limitation of social media is the existence of rumors or stories without any fundamental basis in facts. Sometimes healthcare professionals, scientific researchers, politicians, etc., can make declarations that could seem correct but this “expert” still stepped outside their area of expertise or even use scientific document or research paper that has not been peer-reviewed [23]. 

In addition, but linked with previous limitations, we could not find enough data to track down a source of information that is available to distinguish between valid or invalid information [10,22]. 

## 4. Conclusions

Twitter can be used as a measuring form of public interest or concern about health-related events, which can serve to generate data on emerging time trends for greater effectiveness of actions, interventions and policy responses to avoid misinformation about these diseases and allow communities to play an important role in the fight against and prevention of diseases.

Finally, we must insist that the pillars of the health system and of any epidemiological projection reside in an adequate information and data processing system. We live in a complex and interrelated system where many factors can be decisive in influencing the health status of the population.
